# Genetically proxied HTRA1 protease activity and circulating levels independently predict risk of ischemic stroke and coronary artery disease

**DOI:** 10.21203/rs.3.rs-3523612/v1

**Published:** 2023-11-07

**Authors:** Martin Dichgans, Rainer Malik, Nathalie Beaufort, Koki Tanaka, Marios Georgakis, Yunye He, Masaru Koido, Chikashi Terao, Christopher Anderson, Yoichiro Kamatani

**Affiliations:** LMU Munich; LMU Munich; Institute for Stroke and Dementia Research; The University of Tokyo; LMU Munich; The University of Tokyo; Institute of Medical Science, The University of Tokyo; RIKEN Center for Integrative Medical Sciences; Massachusetts General Hospital; The University of Tokyo

## Abstract

*HTRA1* has emerged as a major risk gene for stroke and cerebral small vessel disease with both rare and common variants contributing to disease risk. However, the precise mechanisms mediating this risk remain largely unknown as does the full spectrum of phenotypes associated with genetic variation in *HTRA1* in the general population. Using a family-history informed approach, we first show that rare variants in *HTRA1* are linked to ischemic stroke in 425,338 European individuals from the UK Biobank with replication in 143,149 individuals from the Biobank Japan. Integrating data from biochemical experiments on 76 mutations occurring in the UK Biobank, we next show that rare variants causing loss of protease function *in vitro* associate with ischemic stroke, coronary artery disease, and skeletal traits. In addition, a common causal variant (rs2672592) modulating circulating HTRA1 mRNA and protein levels enhances the risk of ischemic stroke, small vessel stroke, and coronary artery disease while lowering the risk of migraine and age-related macular dystrophy in GWAS and UK Biobank data from > 2,000,000 individuals. There was no evidence of an interaction between genetically proxied HTRA1 activity and levels. Our findings demonstrate a central role of HTRA1 for human disease including stroke and coronary artery disease and identify two independent mechanisms that might qualify as targets for future therapeutic interventions.

## INTRODUCTION

Stroke and coronary artery disease (CAD) are the leading causes of death worldwide with an increasing burden particularly in low-income countries^1, 2^. Recent genome-wide association studies (GWAS) have identified multiple risk loci for stroke and CAD and demonstrated that genetic information can be harnessed to define drug targets^3, 4, 5^.

High-Temperature Requirement A serine peptidase 1 (*HTRA1*), a known regulator of the extracellular matrix (ECM), has emerged as a risk gene for various phenotypes including stroke^3, 4^ and cerebral small vessel disease (cSVD)^6, 7, 8, 9^. However, the mechanisms mediating this risk remain poorly understood. Rare mutations in *HTRA1* cause a Mendelian syndrome of small vessel stroke further manifesting with lumbago^6, 7, 8, 10, 11, 12^. Using data from the UK Biobank (UKB), we recently showed that rare missense variants targeting the *HTRA1* protease domain are associated with radiological hallmarks of cSVD^13^. Whether such variants associate with stroke or other cardiovascular traits in the general population remains unclear. Aside from rare mutations, common variants at *HTRA1* have been shown to associate with disease risk in GWAS for stroke^3, 4^, CAD^5^, migraine^14^, and age-related macular dystrophy^15^, implying that the consequences of such variants reach beyond brain microvessels. However, the full spectrum of phenotypes associated with genetic variation in HTRA1 and the precise mechanisms mediating disease risk remain elusive.

Biochemical studies on *HTRA1* mutations involved in Mendelian stroke have demonstrated a loss of protease activity^6, 7, 8, 10, 16, 17^. However, there have been no systematic efforts to study the functional consequences of rare variants in the general population and their relationship with disease. Also, it remains unknown whether the associations between common single nucleotide polymorphisms (SNPs) at *HTRA1* and risk for stroke, CAD, and migraine relate to a shared set of causal variants. A third unresolved question relates to the interaction between rare and common variants at *HTRA1* with respect to disease risk.

To address these questions, we initially performed rare-variant aggregation tests for ischemic stroke in the UKB^18^ using a classical case-control design and a family history proxy phenotype^19^ with replication in the Biobank Japan^20^. To assess whether HTRA1 protease activity mediates the observed association with ischemic stroke we determined the consequences of 78 UKB protease domain variants on enzymatic activity *in vitro*. We next performed phenome-wide association studies (PheWAS) with genetically proxied HTRA1 activity as the exposure and uncovered the phenotypic spectrum of *HTRA1* mutation carriers which included stroke-related, CAD-related, and additional traits. Using GWAS data, we performed causal variant analysis on multiple traits and analyzed their association with HTRA1 mRNA and protein levels. As a last step we explored the interactions between genetically proxied HTRA1 activity and levels on cardiovascular risk.

## RESULTS

**Figure 1:**
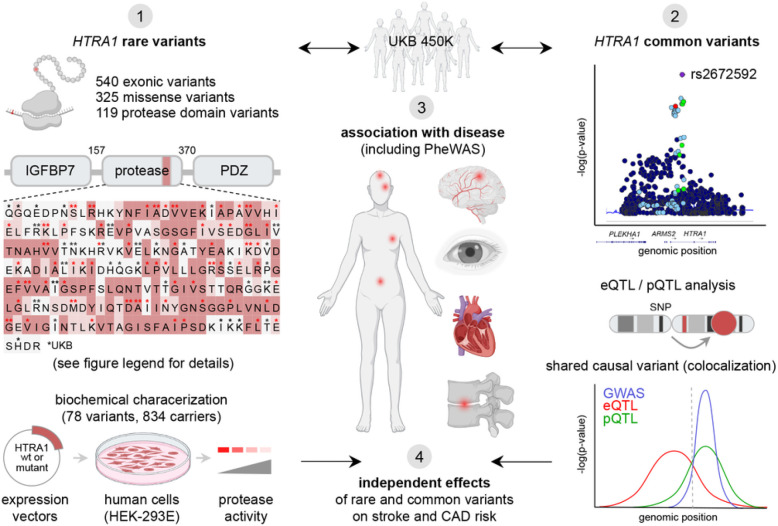
Analytic workflow of human phenotypes linked to rare and common *HTRA1* variants. (1) 78 rare missense variants in the *HTRA1* protease domain were selected for biochemical analysis. Variants targeting residues conserved in human HTRA1–4 were prioritized. Amino acids strictly conserved in 3 or 4 human HTRAs are highlighted in bright or dark red, respectively; asterisks label the 119 missense protease domain variants identified in the UKB; red asterisks mark the 78 selected variants. Protease activity was measured in secretomes from HEK-293E cells transfected to overexpress wt or mutant HTRA1. (2) Common variant assessment included GWAS, eQTL, pQTL and colocalization analyzes. (3) Rare and common *HTRA1* variants were investigated for their associations with human disease through a PheWAS framework. (4) The interaction of rare and common *HTRA1* variants on stroke and coronary artery disease (CAD) risk was determined. HTRA1: High-Temperature Requirement A serine peptidase 1; IGFBP7: Insulin-like Growth Factor-Binding Protein 7; PDZ: Post synaptic density protein (PSD95), Drosophila disc large tumor suppressor (DlgA), and Zonula occludens-1 protein (zo-1, PheWAS: Phenome-Wide Association Study; eQTL: expression Quantitative Trait Loci; pQTL: protein Quantitative Trait Loci.

To comprehensively assess the effects of rare and common genetic variation at *HTRA1* on human disease we chose a four-pronged approach comprising (1) rare exonic variant analyses, including *in vitro* protease activity assays, (2) common variant analyses, including GWAS, colocalization, eQTL and pQTL analyzes, (3) assessment of their association with disease, including phenome-wide association studies (PheWAS) and (4) rare and common variant interaction analysis (Fig. 1).

### Rare exonic variants in HTRA1 associate with ischemic stroke in the general population

To identify putative associations between rare genetic variation in *HTRA1* and ischemic stroke risk in the general population, we used the UKB 450K WES dataset and performed whole-exome rare variant burden analyses using regenie^21^. Given the known involvement of *HTRA1* in Mendelian stroke^6, 7, 8, 10, 11^ and the increase in association power using a liability threshold model, that is conditional on case-control (CC) status and family history (LT-FH)^19^, we employed both a classical CC and LT-FH phenotype.

Rare damaging missense (REVEL score > 0.5) and LoF variants in *HTRA1* (total N = 144 variants; 1,323 carriers) were associated with ischemic stroke both in the LT-FH analysis (p = 5.72E^−7^; effect size: 0.019 [s.e. 0.0038]) and CC analysis (p = 1.51E^−4^, OR = 2.27 [1.56–3.32]). A significant association was also found when restricting the analysis to rare damaging missense variants (N = 118 variants; 1,087 carriers; LT-FH analysis: p = 1.20E^−6^, beta = 0.014 [s.e. 0.0029]; CC analysis: p = 9.83E^−4^, OR = 2.18 [1.44–3.32]). The association was independent of systolic blood pressure, diastolic blood pressure, LDL-cholesterol, HDL-cholesterol, diabetes or BMI in multivariable analysis (LT-FH analysis: p = 3.54E^−6^, standardized beta = 0.13 [s.e. 0.03]). Moreover, the effect size of *HTRA1* rare variant carrier status on the LT-FH phenotype was greater than for any of these risk factors (**Suppl. Figure 1**). To substantiate our findings, we performed identical rare variant aggregation tests in the Biobank Japan (N = 143,149 individuals) and confirmed that rare (6.64E^−5^ < MAF < 0.01) variants in *HTRA1* (4 variants, 194 mutation carriers) are associated with ischemic stroke using the LT-FH phenotype (p-value = 0.026, beta = 0.016 [s.e. 0.0093]) and the CC phenotype (p-value = 0.0061 in the SKAT test with identical effect direction as in burden test). Taken together, these analyses establish rare exonic variants in *HTRA1* as a cause of ischemic stroke in the general population.

### Ischemic stroke related to rare missense variants in HTRA1 is linked to loss of protease activity

Given the molecular function of HTRA1 as a serine protease, we next explored the consequences of rare missense *HTRA1* variants on enzymatic activity as a putative mechanism underlying ischemic stroke risk. We focused on variants within the protease domain (amino acids 157–480) as this domain is a prime target for mutations in *HTRA1*-related familial cSVD^6, 7, 8, 10, 11^. We prioritized mutations that affected residues conserved across all four members of the human HTRA family and selected 78 variants in 834 mutation carriers (all heterozygous except one compound heterozygote carrier) for biochemical assessment (Fig. 1, **left panel**). Specifically, we measured protease activity in HEK-293E cells overexpressing HTRA1 wild type (wt), an active site mutation (S328A), or the selected UKB mutations (**Suppl. Figure 2**).

**Figure 2:**
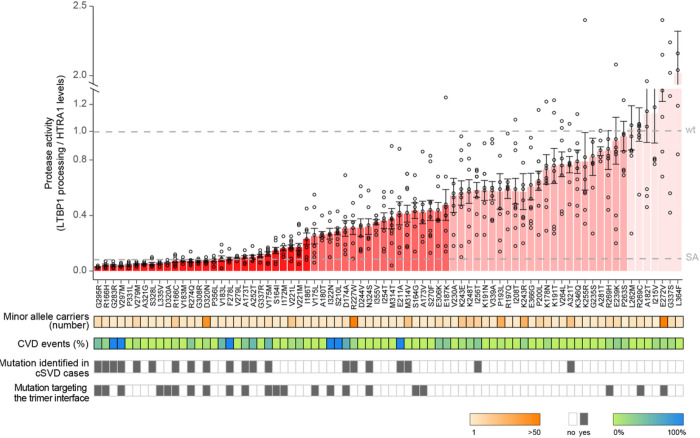
Consequences of rare missense protease domain variants on enzymatic activity. Upper panel: HTRA1 protease activity was measured using LTBP1 as a substrate and corrected for HTRA1 levels. Histogram depicts the average activity +SD measured in 5–10 experiments; circles: data points. The ratio cleaved / intact LTBP1, normalized to HTRA1 levels of wt HTRA1 (set to 1) and of the active site mutant S328A (SA) are marked by dashed lines. Variants were stratified into the following categories: residual activity < 0.25 (dark red; n=28); ≥ 0.25 and < 0.5 (light red; n=18); ≥ 0.5 and < 1.0 (dark pink; n=23), and ≥ 1.0 (light pink; n=7). Lower panels: i) minor allele counts in the UKB), ii) percentage of cardiovascular events (CVD), defined as the combination of ischemic stroke and CAD events) in the UKB, iii) mutations reported in familial and sporadic cSVD cases, and iv) mutations targeting the HTRA1 protomer-protomer interface.

Most variants strongly (residual activity < 0.25; N = 28 variants; N = 131 carriers), moderately (residual activity ≥ 0.25 and < 0.5; N = 18; N = 436 carriers), or mildly (residual activity ≥ 0.5 to < 1.0; N = 23, N = 169 carriers) impaired protease activity compared to wt HTRA1. Seven variants showed a trend towards elevated activity (residual activity ≥ 1.0; N = 97 carriers) (Fig. 2, **upper panel, and Suppl. Table 1**), whereas two variants (G213R and G213V, N = 2 carriers) were not secreted (**Suppl. Figure 3**) and were thus excluded from further analyses. Variants with a residual activity < 0.5 were markedly enriched for mutations previously linked to familial or sporadic cSVD (39% vs 7% in those with residual activity ≥ 0.5, p = 0.002, Fig. 2, **lower panel, and Suppl. Figure 4**).

**Figure 3:**
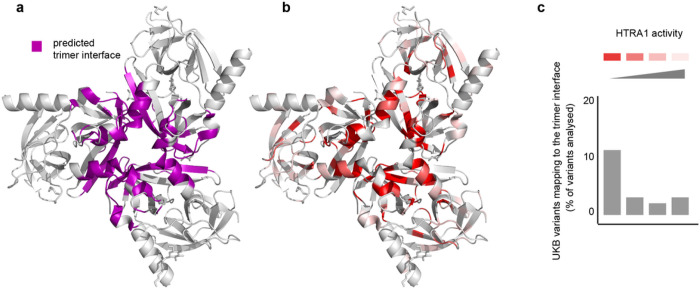
The HTRA1 protomer-protomer interface is a hotspot for severe loss of function mutations. Structure of the HTRA1 trimer (PDB ID 3TJO, catalytic domain of the inactive S328A mutant). **a**, The predicted protomer-protomer interface (PyMOL plugin ‘InterfaceResidues’) is highlighted in purple. **b**, Rare missense variants with experimental protease activity data are highlighted as follows: residual activity < 0.25 (dark red); ≥0.25 and < 0.5 (light red); >0.5 and < 1 (dark pink), and ≥ 1.0 (light pink). **c,** For all identified interface variants, we counted the percentage of mutations belonging to each protease activity category.

To obtain insights into structure-function relationships, we next mapped the position of the 76 variants with available protease activity data onto the structure of the HTRA1 trimer (PDB code 3TJO). We found the predicted trimer interface (Fig. 3a) to be the preferential site for variants showing residual activity < 0.5 (41% vs 10% of those with residual activity ≥ 0.5, p = 0.003) (Fig. 3b and c and Fig. 2, **lower panel**), underscoring the importance of the protomer-protomer interface for HTRA1 activity.

We next correlated the effects of the 76 studied variants on enzymatic activity with their effect sizes for the LT-FH stroke phenotype and found a significant negative correlation (r=−0.35, p = 0.0041) (**Suppl. Figure 5**). Expanding on our previous results^13^, we also found the reduction of protease activity to correlate with the effect sizes for log white matter hyperintensity (WMH) volume (N = 36,681 individuals; N variants = 31; N carriers = 109; r=−0.69, p = 0.0093) (**Suppl. Figure 6**). We further proxied the HTRA1 activity for all UKB individual based on their genotype and the *in vitro* biochemical measurements. Generalized additive model analysis confirmed that the LT-FH stroke phenotype and logWMH volume increased with decreased HTRA1 activity (LT-FH: p = 3.28E^−5^ after correction for sex, age at baseline and 10 PCs; logWMH volume p = 4.79E^−9^ after correction for sex, age at baseline and 10 PCs) (**Suppl. Figures 7 and 8**). Collectively, these data imply that the increase in ischemic stroke risk conferred by rare missense variants in HTRA1 relates to a loss of enzymatic activity.

### PheWAS reveals association between genetically proxied loss of HTRA1 protease function and vascular as well as skeletal traits in the UKB

**Figure 4:**
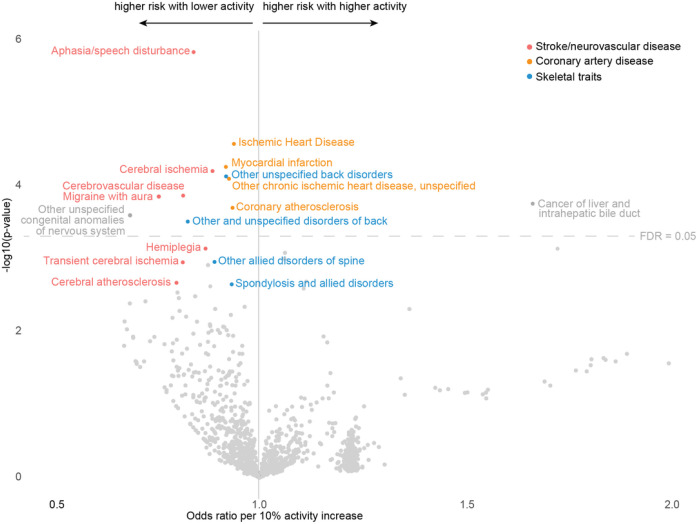
PheWAS of imputed HTRA1 protease activity reveals associations with neurovascular, skeletal and CAD-related traits. PheWAS results using predicted HTRA1 protease activity for each individual as the dependent variable. The x-axis shows the Odds Ratio for a 10% increase in HTRA1 activity, the y-axis displays a log-transformed p-value. The FDR cutoff of 0.05 is presented as a dashed line.

Using the experimentally determined consequences of the 76 variants on HTRA1 activity as an exposure, we next performed a phenome-wide association analysis (PheWAS) to explore associations with all disease outcomes in the UKB. We leveraged DeepPheWAS^22^ to assign case/control status of 1,463 standardized Phecodes for binary disease phenotypes to each individual. For each Phecode, we performed logistic regression using sex, age and 10 PCs as covariates. We deemed associations at an FDR level of 5% to be statistically significant. In addition to neurovascular disease-related Phecodes (P292.1: aphasia/speech disturbance - a frequent manifestation of stroke^23^, TIA^24^, and migraine with aura^25^; P433.3: cerebral ischemia; P433: cerebrovascular disease; P340.1: migraine with aura), we found lumbago- and spondylosis-related Phecodes (P724: other unspecified back disorders; P724.9: other and unspecified disorders of back) to be associated with reduced HTRA1 activity (Fig. 4 and Table 1). Of note, next to stroke and WMHs, lumbago and spondylosis are among the most common manifestations in Mendelian disorders related to *HTRA1* mutations^17, 26^, suggesting that our approach accurately identified key phenotypic traits.

As a major novel finding, we further identified a significant association between reduced protease activity and a range of CAD-related Phecodes including P411 (ischemic heart disease), P411.2 (myocardial infarction), P411.8 (other chronic ischemic heart disease, unspecified) and P411.4 (coronary atherosclerosis). CAD traits have so far not been considered part of the phenotypic spectrum of familial disorders linked to *HTRA1* mutations. The directionality of effects was identical to stroke risk in that rare variants reducing HTRA1 activity were associated with an increased CAD burden. The effect size was strongest for P292.1 (aphasia/speech disturbance: OR = 0.84, CI95 [0.79–0.89] per 10% increase in HTRA1 activity) and overall effect sizes seemed stronger for cerebrovascular compared to CAD. The only phenotype for which a lower HTRA1 activity was associated with a lower risk of was P155 (Cancer of liver and intrahepatic bile duct; OR = 1.67, CI95 [1.37–2.03] per 10% increase in HTRA1 activity). Collectively, these findings show that the consequences of rare loss-of-function variants in *HTRA1* extend beyond known phenotypes and include neurovascular, cardiovascular, and skeletal traits.

### A common causal variant affects HTRA1 mRNA and protein levels and is associated with ischemic stroke and coronary artery disease

**Figure 5:**
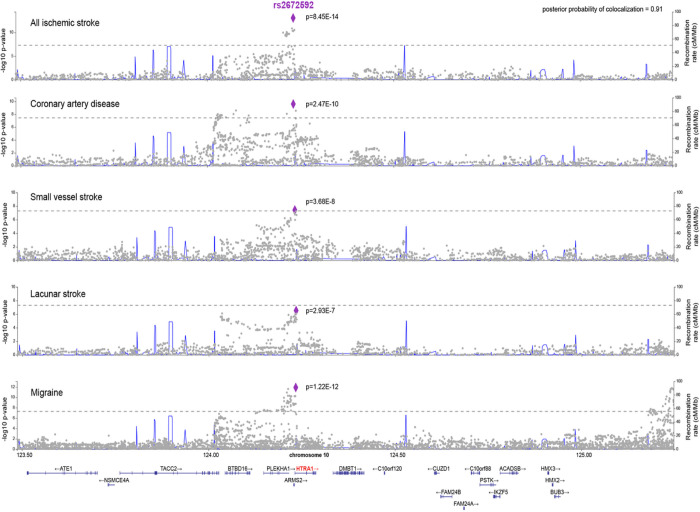
A common variant in *HTRA1* shows colocalization with five neurovascular phenotypes. For each of the phenotypes (any ischemic stroke; coronary artery disease; small vessel stroke; lacunar stroke; migraine) the *HTRA1* gene ±150kb is depicted on the x-axis. The y-axis depicts the −log10 p-value of the respective GWAS. The dashed lines depict genome-wide significance (p=5E^−8^). We used Hyprcoloc to compute the posterior probability of colocalization (PIP) for each SNP in the genomic interval. The highest PIP was estimated for rs2672592. For this SNP, we depict the p-value for each of the five phenotypes.

Previous GWAS found common variants in *HTRA1* to be associated with any ischemic stroke, small vessel stroke, CAD, and migraine^3, 4, 5, 14, 27^. To investigate the possibility that a single variant or limited set of variants is responsible for these associations with multiple traits, we performed colocalization tests using the latest GWAS summary statistics for ischemic stroke, small vessel stroke, lacunar stroke, CAD, and migraine, within a window of ± 150kb from *HTRA1* using Hyprcoloc^28^. A common variant (rs2672592, G/T, allele frequency of the minor T allele = 36%) in the first intron of *HTRA1* was the single causal variant for all tested phenotypes (posterior probability of colocalization = 0.91). The major G allele was associated with increased risk for ischemic stroke, small vessel stroke, lacunar stroke and CAD while being associated with a lower risk for migraine (Fig. 5). Of note, rs2672592 is a known eQTL and pQTL for *HTRA1* in peripheral blood mononuclear cells and plasma, with the G allele being associated with lower mRNA and protein levels^29, 30^. The association between rs2672592 and the above disease and expression phenotypes was further confirmed using Primo^31^ (posterior probability = 0.99, FDR = 0.03), making this SNP the prime candidate to be the common causal variant at this locus and to influence disease risk via modulation of both HTRA1 mRNA and protein levels (**Suppl Fig. 9**).

We further performed a phenome-wide association study (PheWAS) using imputed allele dosage of rs2672592 as an exposure and sex, age and 10 PCs as covariates. We confirmed associations with cardiovascular disease (CVD), including both stroke and CAD-related Phecodes in the UKB (Table 2). Specifically, we found coronary atherosclerosis (P411.4) and occlusion and stenosis of precerebral arteries (P433.1) to be among the top signals. Similar to the effect of rare loss-of-protease-function variants, stronger effects were found for cerebrovascular disorder (P433.1: OR for allelic dosage of the major G allele = 1.10 [1.05–1.16]) than for cardiovascular disorders (P411.4: OR for allelic dosage of the major G allele = 1.05 [1.04–1.07]). Of interest, associations with Phecodes relating to age-related macular degeneration (AMD) and other retinal disorders showed opposite directionality compared to CVD-related phenotypes in that risk was higher in carriers of the minor T allele. Collectively, these findings show a common causal variant affecting HTRA1 mRNA and protein levels is associated with ischemic stroke and CAD.

### Genetically proxied HTRA1 activity and levels independently predict risk of stroke and coronary artery disease

To scrutinize the relationship between genetically proxied HTRA1 activity and levels in mediating disease risk, we next performed logistic regression analyses. In an interaction model with sex, age and 10 PCs as covariates there was no indication for an interaction between HTRA1 activity and HTRA1 mRNA/protein levels with regard to the association with stroke or CAD (interaction p-value > 0.5 for both phenotypes tested). These results show that the effects of HTRA1 activity as determined by rare damaging missense variants within the protease domain and HTRA1 mRNA and protein levels as proxied by rs2672592 on cardiovascular risk are independent.

**Figure 6:**
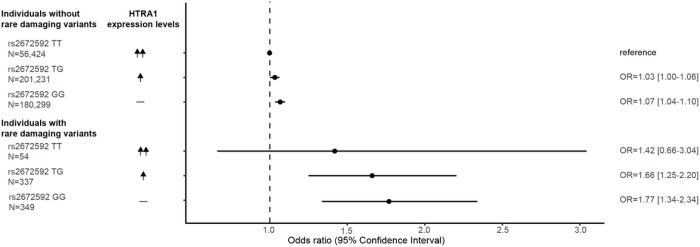
Genetically proxied HTRA1 activity and levels independently predict risk of stroke and coronary artery disease. Forest plot for each of the strata defined by rs2672592 genotype (GG, TG, or TT) and presence of a rare damaging variant. Cardiovascular phenotype (CVD): pooled ischemic stroke and CAD events.

Finally, to explore the combined effect of HTRA1 activity and levels on population health we combined ischemic stroke and CAD into a CVD phenotype and stratified patients into disease risk categories as predicted by HTRA1 activity and levels. We found individuals with reduced HTRA1 activity predicted by a rare protease domain variant (N = 349) in combination with a GG genotype at rs2672592 to have an OR of 1.77 [1.34–2.34] for developing CVD compared to the reference group (TT genotype and no damaging rare variant; N = 56,424) (Fig. 6).

## DISCUSSION

Our findings highlight the importance of HTRA1 for human disease and identify two independent mechanisms by which rare and common variants at *HTRA1* affect cardiovascular risk. We show that rare variants in *HTRA1* are linked to ischemic stroke in the general population and that variants causing loss of protease activity associate with ischemic stroke, CAD, and skeletal traits. We further show that a common causal variant (rs2672592) modulating circulating *HTRA1* mRNA and protein levels enhances the risk of ischemic stroke, small vessel stroke, and CAD while lowering the risk of migraine and age-related macular dystrophy. Importantly, there was no evidence of an interaction between the effects of genetically proxied HTRA1 activity and levels on CVD risk suggesting that future strategies addressing either mechanism could be used to fine-tune the phenotypic consequences of therapeutic interventions.

Cardiovascular risk factors have repeatedly been reported in *HTRA1*-related familial stroke^17^. We found the association of rare damaging missense and loss-of-function variants in *HTRA1* with ischemic stroke to be independent of traditional risk factors. Recent work has shown that loss of HTRA1 function results in profound changes of the vascular matrisome^16, 32, 33^. HTRA1 cleaves multiple constituents of the ECM including latent TGFβ binding protein (LTBP) 1 and 4, fibronectin (FN), vitronectin (VN) and clusterin (CLU)^16, 32, 33, 34, 35, 36^. Consequently, disease-related loss of HTRA1 function leads to accumulation of these and other matrisomal proteins in the brain vasculature^7, 32, 33^. Alterations of the ECM take center stage in cSVD^37, 38^ as further evidenced by the causal role of mutations in COL4A1^39, 40^, COL4A2^41, 42^, and LAMB1^43^. Extending these observations, the association between rare variants in *HTRA1* and risk of CAD identified here suggests a mechanistic overlap between small vessel and large artery disease that involves multiple proteins of the ECM^26, 44^.

So far, the loss of HTRA1 activity and its relationship with disease have been regarded as a binary trait. Our results demonstrate the importance of considering HTRA1 activity as a continuous phenotype. For one, we show that naturally occurring mutations in *HTRA1* result in variable degrees of enzymatic activity covering the full range from complete loss of function to increased activity, as exemplified by L364F. We further demonstrate a gradual increase in the risk of ischemic stroke, CAD, and skeletal traits with larger reductions in genetically proxied protease activity. Whether residual protease activity also influences the penetrance of mutations and clinical severity remains to be determined. Mapping *HTRA1* variants to the HTRA1 structure identified the trimer interface as a hotspot for the most deleterious variants with respect to enzymatic function and cardiovascular risk. This could open perspectives for variant severity prediction via computational approaches.

Reduced HTRA1 protein levels should result in reduced protease activity. Accordingly, the phenotypes associated with genetically proxied HTRA1 protease activity and levels exhibit overlap with respect to cardiovascular risk. Of note, however, we found no association between low HTRA1 activity and risk for retinal disorders, and between low HTRA1 levels and skeletal traits. Also, genetically proxied lower activity was linked to higher risk for migraine with aura, whereas genetically proxied lower levels protected against migraine. Several explanations come in mind: First, in addition to their effects on protease activity, some missense variants might interfere with mRNA or protein levels e.g., by influencing their stability or protein folding. Second, HTRA1 exhibits functions related to protein quality control that are unrelated to protease activity^45^: HTRA1 disintegrates Tau and AB fibrils in a PDZ-dependent but protease-independent manner which is then followed by proteolytic degradation. Whether a similar dual mechanism also applies to other proteins is currently unknown. Third, the observed divergence on phenotype associations could be explained by tissue- or cell type-specific effects. For instance, in the brain, HTRA1 is predominantly produced by astrocytes, followed by arterial endothelial cells, whereas Müller and retinal pigment epithelial cells are the predominantly expressing cell types in the retina^46, 47^. Also, the effects of rs2672592 and the repertoire of HTRA1 substrates^33, 35, 36, 48, 49^ might vary between cell types.

Our findings open perspectives for risk stratification through mutational screening in combination with measurements of protein function and levels. Our findings further have implications for future therapeutic strategies. In principle, cardiovascular risk could be lowered either by restoring HTRA1 activity or by increasing its levels. However, and as shown by our PheWAS, raising HTRA1 levels is predicted to result in an increased risk for AMD and other retinal disorders. Conversely, raising HTRA1 activity is predicted to increase the risk of liver and bile duct cancer. Together, this highlights the need to develop organ or cell-type specific therapeutics. Intravitreal administration of an inhibitory HTRA1 antibody is currently being tested in a clinical trial for geographic atrophy secondary to AMD^35, 50^ and there are various strategies to specifically target individual organs including brain and heart^51^ or cell types such as macrophages^52^.

Our study has limitations. Although we assessed the functional consequences of 76 missense protease domain variants on enzymatic activity, there remain numerous other variants that warrant scrutiny, particularly additional missense variants in the predicted trimer interface, or targeting the signal, IGFBP7 or PDZ domain, as well as predicted LoF variants. Also, we used HEK-293E cells and LTBP1 as the sole cell line and substrate in our biochemical assays, while HTRA1 protease activity could vary depending on the cell-type or substrate. Moreover, the statistical power of PheWAS remains low for certain Phecodes, and we thus might have missed associated phenotypes, emphasizing the need for larger sample sizes.

In conclusion, we demonstrate associations between rare and common genetic variation in *HTRA1* and cardiovascular outcomes in the general population. We identify two independent mechanisms by which these variants affect cardiovascular risk. Our findings imply, that future clinical applications would need to consider both HTRA1 activity and levels. They further demonstrate a mechanistic overlap between cerebral small vessel and large artery disease and a pivotal role of the ECM.

## Methods

### UK Biobank

Our primary dataset consists of ~450,000 individuals from the UK Biobank (UKB) with available WES data and linked health records. We only included unrelated (KING coefficient < 0.177 corresponding up to 2^nd^ degree) individuals of European origin. To assign European ancestry, we used flashpca2^53^ and constructed 10 principal components (PCs) for all UKB participants and participants in 1000 Genomes v3 data using ~20,000 SNPs as input. With the resulting PCs, we trained a random forest classifier based on the gold standard 1000G v3 ethnicities. We combined samples from CEU, TSI, GBR, and IBS into a European clade. Each UKB individual was classified using the random forest classifier and those having a probability of >95% of being European were put in the European ancestry. Descriptive statistics of the studied individuals are provided in **Suppl. Table 2**.

### Phenotype definitions

We ascertained ischemic stroke status based on hospital episode stay (HES) data, death certificates and self-report using published ICD10 codes (I63-I63.9, I64.X)^54^. Focusing on the European subset of the UKB, we identified 8,557 cases with any ischemic stroke (AIS) and 415,947 controls. This information was used to construct a classical case/control (CC) phenotype. Non-ischemic stroke cases were removed from the analysis. We used published definitions for coronary artery disease (ICD10: I21, I22, I23, I24, I25.1, I25.2, I25.5, I25.6, I25.8, I25.9, OPCS4: K40, K41, K42, K43, K44, K45, K46, K49, K50.1, K50.2, K50.4, K75)^55^.

### LT-FH phenotype

The UKB holds information on paternal, maternal or sibling stroke (coded in UKB fields *20107, 20110* and *20111*, respectively) and the number of siblings (UKB fields *1883* and *1873*). We used this information to construct the LT-FH phenotype as previously described^19^. Specifically, we used information on proband stroke, paternal stroke, maternal stroke, sibling stroke and number of siblings (with a maximum of 5 considered) to construct the continuous LT-FH phenotype. We used a heritability of 0.17 as proposed in the original publication^19, 56^. While the UKB family history fields do not distinguish between ischemic and non-ischemic stroke, we considered a family history of any stroke as being equivalent to ischemic stroke.

### Rare variant burden test

For the rare variant aggregation tests, we used regenie^21^. Due to low power in single variant association tests, we used gene aggregation burden as our primary outcome. To create rare variant aggregation masks, we used five different minor allele frequency (MAF) bins (<0.01, <0.001, <0.0001, <1E^−5^, singletons).

We further determined the functional consequences of exome-wide variants using the Variant Effect Predictor (VEP) tool v101^57^. For the whole-exome burden test on stroke analysis, we selected rare variants (MAF < 0.01) that are either predicted to be damaging by REVEL^58^ (REVEL score > 0.5) or predicted to exert a high-confidence loss-of-function (LoF) effect using the LoFTEE^59^ plugin in VEP. We used the GrCh38 refFlat definition of genes as provided by the UCSC genome annotation database. In regenie, we used all members of the burden, SKAT^60, 61^ and ACAT^62^ families with sex, age and 10 PCs as covariates. We deemed p-values < 2.5E^−6^ as being significant, corresponding to a Bonferroni correction for 20,000 genes.

### Replication in Biobank Japan

We sought to replicate the association of a rare variant burden in HTRA1 and stroke in the Biobank Japan also using a CC and LT-FH approach^20^. BioBank Japan Project was a multi-institutional hospital-based biobank that collected DNA and clinical information from about 200,000 patients with 47 common diseases between 2003 and 2007 (1st cohort). The 180K Participants were genotyped with the Illumina HumanOmniExpressExome BeadChip or a combination of the Illumina HumanOmniExpress and HumanExome BeadChip. Quality control of participants and genotypes was performed following the previous study^63, 64^. After pre-phasing without an external reference using EAGLE v2.4.1^65^, the genotype data for individuals of the East Asian ancestry were imputed with JEWEL3k (3,256 Japanese whole-genome sequence data (WGS) + 2,504 1000G Phase3 v5) reference panel^66^ using Minimac4 v1.0.2^67^. Imputed variants with Rsq < 0.3 were removed and the coordinates were lifted from hg19 to hg38 using GWASLab v3.3.10^68^. For both CC and LT-FH approaches, we excluded individuals who lacked family history information. For cases, we ascertained ischemic stroke status via interviews and reviews of medical records using a standardized questionnaire. Individuals without ischemic stroke, cerebral aneurysm, intracerebral hemorrhage, subarachnoid hemorrhage, or unruptured cerebral aneurysm were selected as controls. We identified 18,865 cases with any ischemic stroke (AIS) and 124,284 controls. For LT-FH phenotype, we further used the information on paternal or maternal ischemic stroke status from interviews of medical records. This status for siblings was not available in Biobank Japan. Rare variant burden test was conducted using the same methods as in UKB.

### Biochemical characterization of UKB variants

#### Variant selection:

Focusing on missense variants targeting the HTRA1 protease domain (amino acids 157–370), UKB variants were selected for biochemical assessment based on residue conservation in human HTRAs according to Clustal Omega^69^. Specifically, all variants strictly conserved in human HTRA1–4 (n= 48), 29 out of 30 variants conserved in 3 human HTRAs (1 variant not included in the HTRA1 trimer structure and thus not selected), and 2 out of 42 variants conserved in ≤ 2 human HTRAs including R227W, the most frequent exonic mutation in the UKB, were selected for analysis (Figure 1).

#### Protease activity measurements:

Eukaryotic expression plasmids encoding either full-length human HTRA1 (aa 1–480) fused to a C-terminal Myc-(His)_6_ tag or the N-terminal region of human LTBP1s (aa 1–689) fused to a C-terminal V5-(His)_6_ tag have been previously described^13, 16^. Mutagenesis was conducted using the QuickChange Lightning Site-Directed Mutagenesis kit (Agilent Technologies).

Human embryonic kidney (HEK-293E) cells were grown in Dulbecco’s modified Eagle’s medium (DMEM) containing GlutaMAX, 10% (v/v) fetal calf serum (FCS), 100 U/ml penicillin and 100 μg/ml streptomycin (all from Invitrogen) at 37°C and 5% CO_2_. Cells were seeded in poly-L-lysine (Invitrogen) coated 6-well-plates, transfected with Lipofectamine 2000 (Invitrogen), and maintained in serum-free medium for 48 h. Secretomes containing the protein of interest were collected and centrifuged for 10 min at 400 *g* to remove debris. Where indicated, cells were lysed in 50 mM Tris, 150 mM NaCl, 1% (v/v) triton X-100, 1% (w/v) sodium deoxycholate, 0.1% (w/v) sodium dodecyl sulphate, pH 7.4 for 20 min at 4°C and samples centrifuged 20 min at 16,000 g. Secretomes from non-transfected cells and from cells overexpressing wild type (wt) HTRA1 or the inactive variant S328A were included in each run as controls.

HTRA1 levels were evaluated by anti-Myc immunoblot (Santa Cruz Biotechnology, #sc-40, 1:2,500) using horseradish peroxidase-coupled secondary antibodies (Dako), immobilon Western kit reagents (Millipore) and the Fusion FX7 documentation system (Vilbert Lourmat). Actin (Sigma-Aldrich Ab #A2066; dilution 1:500) served as loading control for the analysis of cell lysates.

Secretomes from cells overexpressing LTBP1 (5 μl) were exposed to secretomes from non-transfected cells, or from cells overexpressing HTRA1 (20 μl) for 24 h at 37°C and samples were analyzed by anti-V5 immunoblot (Invitrogen, #R960–25, 1:10,000). Where appropriate, wt or mutant HTRA1-containing secretome was diluted in secretome from non-transfected cells to measure HTRA1 activity in samples containing comparable HTRA1 concentrations. Datapoints exhibiting low residual intact LTBP1 or low mutant HTRA1 levels compared to wt HTRA1 (see Source Data) were excluded.

LTBP1 and HTRA1 signals were quantified using ImageJ. LTBP1 processing was evaluated as the ratio cleaved / intact LTBP1 and HTRA1 protease activity was estimated as the ratio LTBP1 processing / HTRA1 levels. The activity of wt HTRA1 was set to 1. Activity thresholds for follow-up analyses were arbitrarily set as follows: (i) activity estimates <0.25, (ii) >0.25 to <0.5, (iii) >0.5 to <1.0 and (iv) >1.

### Trimer interface prediction

We used the PyMOL plugin “InterfaceResidues.py” (https://pymolwiki.org/index.php/InterfaceResidues) to predict the HTRA1 trimer interface.

### Correlation of effects

We constructed Pearson correlation coefficients between (i) effect sizes from single-variant analyses of logWMH (white matter hyperintensity) volume in the UKB vs. effect sizes constructed from the average HTRA1 activity ratio and (ii) effect sizes from single-variant analyses of LT-FH the UKB vs. effect sizes constructed from the average HTRA1 activity. All analyses were performed using R 4.3.0.

### Predicting individual HTRA1 activity

To assess the association of HTRA1 activity with cardiovascular endpoints, we genetically proxied HTRA1 activity for each individual in the UKB. For this, we set HTRA1 activity for individuals without a rare HTRA1 protease domain mutation to 100%. For mutation carriers we multiplied the measured average HTRA1 activity from the *in-vitro* assays with 100%. This activity estimate was used as an exposure in our analysis. We used Generalized Additive Models (GAM) to show association between this exposure and the LT-FH phenotype and logWMH volume, respectively.

### PheWAS

To explore the association of predicted HTRA1 activity with the full range of phenotypes encoded in the UKB, we used DeepPheWAS to assign participants to standardized Phecodes.^22^ We used all ICD10 codes (main position, secondary position, death records) from the UKB. We excluded Phecodes with <100 cases and Phecodes that are male- or female-specific. Individuals were assigned a case status if >1 ICD10 code mapped to the respective Phecode. Individuals meeting the pre-specified exclusion criteria were removed from the analysis, otherwise the individual was assigned a control status. We used logistic regression with age, sex and 10 PCs as covariate to test rare variant carrier status (0/1) against the phenotype of interest. Results with p<0.05 were corrected with Firth’s correction. We considered results with FDR<5% as statistically significant.

### Colocalization of common variants

We obtained summary statistics for any ischemic stroke and small vessel stroke from the GIGASTROKE consortium^4^, for coronary artery disease (CAD) from the CARDIoGRAMplusC4D Consortium^5^, for lacunar stroke from the UK DNA Lacunar Stroke studies 1 and 2 and from collaborators within the International Stroke Genetics Consortium^27^, and for migraine through the International Headache Genetics Consortium (IHGC)^14^. We used an interval of +−150kb from *HTRA1* and included all SNPs that were available for analysis for all phenotypes. We used Hyprcoloc^28^ and synchronized effect sizes and standard errors to perform colocalization and report the resulting posterior probability of colocalization.

Blood eQTL and pQTL data were obtained from the eQTLgen consortium^29^ and deCODE^30^, respectively. We used Primo^31^ to perform pleiotropy analyses between eQTL, pQTL and disease phenotype data and report posterior probability of pleiotropy. We used an interval of +−150kb from rs2672592 and included all SNPs that were available for analysis for all phenotypes.

### Stratification of individuals into disease risk classes

Using imputed genotype dosage of rs2672592 and rare HTRA1 protease domain carrier status, we used logistic regression with age, sex and 10 PCs as covariates. We set the lowest risk category (rs2672592 TT and no rare variant) as the reference category and all odds ratios are reported compared to this category.

### Display items

Figures were prepared with R version 4.3.0, ggplot2, Pymol, Adobe Illustrator, Microsoft Power Point and BioRender.com.

## Figures and Tables

**Table 1:** PheWAS results for genetically proxied HTRA1 protease activity in the UK Biobank. Odds Ratios are presented for a 10% increase in HTRA1 protease activity. CI95: 95% confidence interval; FDR: false discovery rate.

Phecode	Phenotype	Odds Ratio	CI95	p-value	FDR
P292.1	Aphasia/speech disturbance	0.84	0.79–0.89	1.46E-06	0.002
P411	Ischemic Heart Disease	0.94	0.91–0.97	2.65E-05	0.015
P411.2	Myocardial infarction	0.92	0.89–0.95	5.48E-05	0.015
P433.3	Cerebral ischemia	0.89	0.84–0.93	6.25E-05	0.015
P433	Cerebrovascular disease	0.92	0.88–0.95	7.40E-05	0.015
P411.8	Other chronic ischemic heart disease, unspecified	0.93	0.89–0.96	7.98E-05	0.015
P724.9	Other unspecified back disorders	0.81	0.75–0.89	1.36E-04	0.020
P340.1	Migraine with aura	0.75	0.68–0.84	1.40E-04	0.020
P155	Cancer of liver and intrahepatic bile duct	1.67	1.37–2.03	1.75E-04	0.023
P411.4	Coronary atherosclerosis	0.93	0.90–0.97	2.00E-04	0.024
P752.2	Other specified congenital anomalies of nervous system	0.68	0.60–0.78	2.55E-04	0.028
P724	Other and unspecified disorders of back	0.83	0.76–0.90	3.08E-04	0.031

**Table 2:** PheWAS results for allelic dosage of the rs2672592 major G allele in the UK Biobank. CI95: 95% confidence interval; FDR: false discovery rate.

Phecode	Phenotype	Odds Ratio	CI95	p-value	FDR
P362	Other retinal disorders	0.86	0.83–0.88	<2.2E-16	<2.2E-16
P362.2	Degeneration of macula and posterior pole of retina	0.80	0.77–0.82	<2.2E-16	<2.2E-16
P362.29	Macular degeneration (senile) of retina NOS	0.80	0.77–0.82	<2.2E-16	<2.2E-16
P411.4	Coronary atherosclerosis	1.05	1.04–1.07	4.42E-10	1.45E-07
P411	Ischemic Heart Disease	1.04	1.03–1.05	2.13E-08	5.59E-06
P411.8	Other chronic ischemic heart disease, unspecified	1.05	1.03–1.07	4.42E-08	9.67E-06
P411.3	Angina pectoris	1.05	1.03–1.07	5.91E-08	1.11E-05
P366	Cataract	0.97	0.95–0.98	5.13E-07	8.42E-05
P411.2	Myocardial infarction	1.05	1.03–1.07	1.09E-06	1.60E-04
P578.8	Hemorrhage of rectum and anus	1.05	1.02–1.07	6.07E-05	0.008
P363	Chorioretinal inflammations, scars, and other disorders of choroid	0.80	0.71–0.89	9.02E-05	0.010
P578	Gastrointestinal hemorrhage	1.03	1.02–1.05	9.03E-05	0.010
P366.2	Senile cataract	0.96	0.95–0.98	9.45E-05	0.010
P433.1	Occlusion and stenosis of precerebral arteries	1.10	1.05–1.16	1.17E-04	0.011
P454.1	Varicose veins of lower extremity	0.96	0.94–0.98	5.46E-04	0.048

## Data Availability

The data that support the findings of this study are available in the manuscript and its online supplements or from the corresponding authors on reasonable request.
